# Faster monitoring of the invasive alien species (IAS) *Dreissena polymorpha* in river basins through isothermal amplification

**DOI:** 10.1038/s41598-021-89574-w

**Published:** 2021-05-13

**Authors:** Joana Carvalho, Alejandro Garrido-Maestu, Sarah Azinheiro, Pablo Fuciños, Jorge Barros-Velázquez, Ramón J. De Miguel, Verónica Gros, Marta Prado

**Affiliations:** 1 Food Quality and Safety Research Group, International Iberian Nanotechnology Laboratory (INL), Av. Mestre José Veiga s/n, 4715-330 Braga, Portugal; 2 Department of Analytical Chemistry, Nutrition and Food Science, School of Veterinary Sciences, University of Santiago de Compostela, Santiago de Compostela, Spain; 3 Department of Zoology, University of Cordoba, Córdoba, Spain; 4 Guadalictio S.L., Córdoba, Spain; 5 Confederación Hidrográfica del Guadalquivir, Ministerio para la Transición Ecológica y el Reto Demográfico, Sevilla, Spain

**Keywords:** Ecology, Environmental sciences, Natural hazards, Freshwater ecology, Invasive species, Analytical biochemistry

## Abstract

Zebra mussel (*Dreissena polymorpha*) is considered as one of the 100 most harmful IAS in the world. Traditional detection methods have limitations, and PCR based environmental DNA detection has provided interesting results for early warning. However, in the last years, the development of isothermal amplification methods has received increasing attention. Among them, loop-mediated isothermal amplification (LAMP) has several advantages, including its higher tolerance to the presence of inhibitors and the possibility of naked-eye detection, which enables and simplifies its potential use in decentralized settings. In the current study, a real-time LAMP (qLAMP) method for the detection of *Dreissena polymorpha* was developed and tested with samples from the Guadalquivir River basin, together with two real-time PCR (qPCR) methods using different detection chemistries, targeting a specific region of the mitochondrial gene cytochrome C oxidase subunit I. All three developed approaches were evaluated regarding specificity, sensitivity and time required for detection. Regarding sensitivity, both qPCR approaches were more sensitive than qLAMP by one order of magnitude, however the qLAMP method proved to be as specific and much faster being performed in just 9 min versus 23 and 29 min for the qPCR methods based on hydrolysis probe and intercalating dye respectively.

## Introduction

*Dreissena polymorpha* is a small freshwater bivalve mollusk commonly known as zebra mussel due to the striped pattern of its shell. It is an invasive alien species (IAS), which are non-native species deliberately or accidently introduced outside their natural habitat by human action, posing a real threat to the biodiversity, human health, or economy^1^. *D. polymorpha* is native to the Black, Caspian, and Azov seas (Ponto-Caspian region) but has already spread throughout Europe and has been quickly spreading throughout the waterways of the United States and Canada^2^. This species was first discovered in Spain in 2001 at the Ribarroja Reservoir (Ebro River)^3,4^. Since its introduction, it has invaded a great part of the Ebro River basin and widely extended to other rivers and lakes. In 2009 it was found in the Guadalquivir River basin, namely at Los Bermejales Reservoir (Granada). Then, in 2011 it invaded the Iznájar Reservoir and in 2015 it was found at La Breña (both in Córdoba province).

The rapid spreading of *D. polymorpha* is in mainly due to their high reproductive capacity and their wide range of tolerance to environmental conditions^5^. A female zebra mussel may release over 40,000 eggs in a reproductive cycle and up to one million in a spawning season. The eggs are released into the water where the fertilization takes place and, within 3–5 days, develop into free-swimming larvae called veligers, which can be transported over long distances by water currents and are the most invasive form of *Dreissena*^6^. After 2–3 weeks, the veligers begin their juvenile stage by settling down, due to the weight of their forming shells, and attaching to firm underwater surfaces using byssal threads^7,8^. After one year of growth, they are already able to reproduce and, as adults, they can even survive 8–10 days out of water, under cool and humid conditions^7,9^. This fast spreading and growing of *D. polymorpha* prevents the few natural predators of this species from causing a steady and long-term decline in their population levels^10^. When attached to hard surfaces, zebra mussels can reach densities as high as 700,000 individuals/m^2^, causing several economic and environmental problems^4,7,11^. This invasive species is capable of clogging intake pipes, grids, pumping systems and other infrastructures, as well as covering hydraulic turbines, ship motors and hulls^7^. They can also attach to native crustaceans, snails and other bivalves, forming dense clusters and limiting their ability to move, feed, reproduce, and eventually leading to the death of these native species^5,12^. In addition, each individual can filter up to one liter of water per day and, due to their high densities, it can strongly affect and change the habitat they are invading^5^, leaving less food for other organisms and contributing to the proliferation of aquatic plants by increasing the transparency of the water. *D. polymorpha* is considered by the International Union for Conservation of Nature (IUCN) as one of the 100 most harmful IAS in the world^13^.

There are several strategies to control zebra mussels in industrial facilities, including mechanical, chemical and biological methods^14,15^. Although these strategies can help eliminating the invasion temporarily, they sometimes require long shutdowns which can be very costly. In addition, these control methods cannot be directly applied to the colonized reservoirs, lakes or rivers, which remain an active point for the species. Therefore, research is gradually turning towards treating the problem in the natural aquatic environment^16^. However, when this species is already dispersed over a large area, its dispersion can only be slowed down by management measures to prevent further spread because, once established, it is very difficult to remove, as it is the case in Spain^17^. Some preventive measures currently applied to avoid the spread and dispersion of *D. polymorpha* include information and awareness campaigns, control and restrictions on navigation and installation of disinfection stations close to the affected water bodies^3^.

The implementation of effective monitoring strategies allowing the detection of this species before its establishment can prevent the spread to other water bodies. In the case of settings isolated from natural ecosystems, the early detection of zebra mussels can increase the success and reduce the costs of operations aimed to remove them. Therefore, there is an urgent need of developing methods for the early detection of invasive species^18^. Traditional methods relying on capture for visual inspection and identification of specimens have limitations, especially regarding the correct identification of closely related species^19^. Moreover, the sampling protocols typically used have low probability of detecting species unless the population density is already high^20^. Molecular identification of species through the analysis of environmental DNA (eDNA) has been used to detect invasive species with greater sensitivity than traditional methods^21–25^. These methods provide more accurate species distribution data and can distinguish between different invasive mussel species^26^, also having the potential to dramatically improve the ability for an early detection and monitoring^18,19,27^.

Traditionally, PCR has been used for the detection of eDNA. However, novel isothermal amplification techniques have emerged in recent years with the goal of providing an analytical solution to some of the drawbacks associated with PCR/qPCR, especially due to their simplicity and reduced thermal budget^28^, which are interesting features for the development of methods for a potential in situ analysis. Among them, loop-mediated isothermal amplification (LAMP) has become the most popular technique^29–31^. Its main advantages compared to PCR include being performed at constant temperature, higher specificity and the possibility for achieving naked-eye detection through turbidity observation^32,33^. The detection can also be performed using intercalating dyes for fluorescence measurement in real-time assays^34,35^.

In this work, a loop-mediated isothermal amplification (LAMP) method was developed for the detection of *D. polymorpha* in environmental water samples. As target, the cytochrome c oxidase sub 1 (COI) was selected. The COI is a mitochondrial gene which increases the possibility of an early detection of the presence of the IAS in the environment due to the fact that mtDNA is present in thousands of copies per cell^36^, at the same time, the COI marker has been identified as the marker of choice for species discrimination^37,38^ . There has been a worldwide effort through the bar-code of life project (www.barcodinglife.org), to characterize all living animals providing unevaluable information for species identification and therefore a large database.

Despite of its advantages, LAMP has not been explored for detection of *D. polymorpha* in environmental samples and, considering the higher complexity of this amplification reaction, it is very important to carefully evaluate the method in order to ensure its *fitness-for-purpose,* particularly in terms of sensitivity and specificity, in comparison with other techniques such as real-time PCR (qPCR) before implementing this technique as part of an strategy for early warning on decentralized settings. Therefore, the current study aims to evaluate the performance of a real-time fluorescence LAMP-based method (qLAMP) developed for the detection of *D. polymorpha* in water samples from the Guadalquivir River basin. In addition, the results obtained were compared with two qPCR approaches, using different detection chemistries (an intercalating dye and a hydrolysis probe), also developed for this study.

## Results

### Optimization of qLAMP

**Figure 1:**
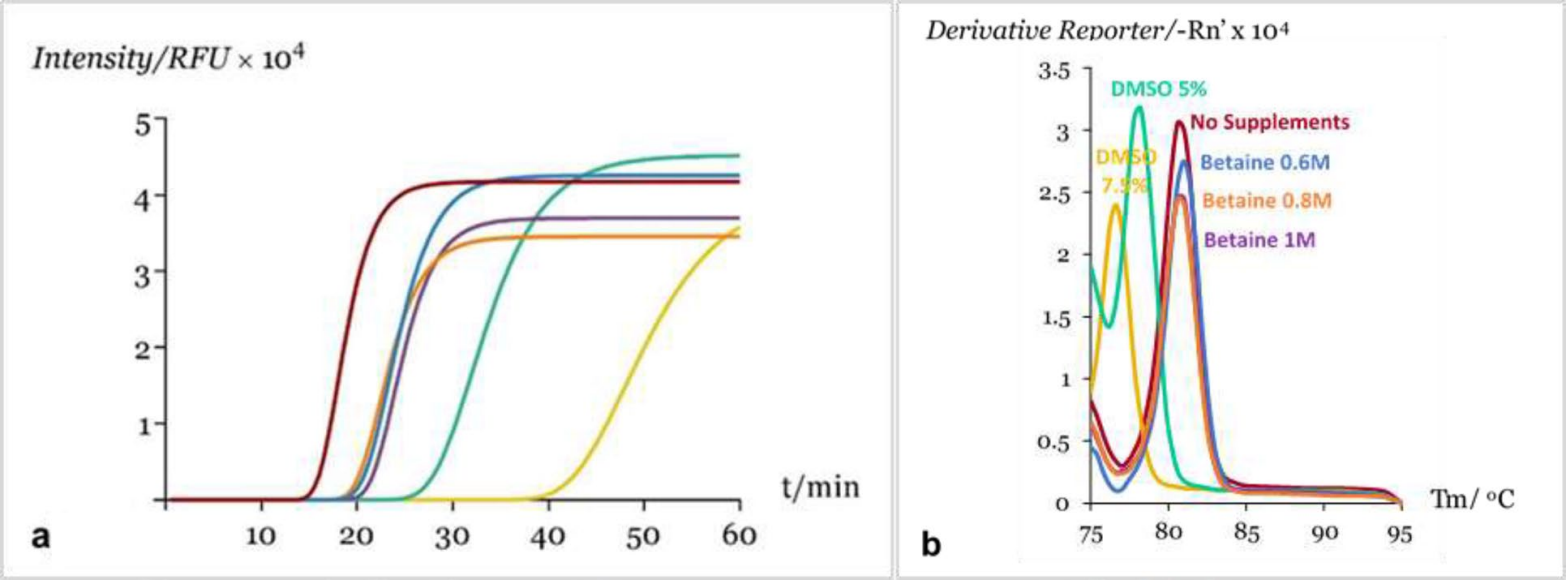
Effect of supplement addition on the qLAMP method. (**a**) qLAMP kinetics in pure *D. polymorpha* DNA (C+) according to the fittings from Eq. (1) performed using GraphPad Prism version 8.0.0 for Windows, GraphPad Software, San Diego, California USA, www.graphpad.com: (deep red line) “C+ No supplement”, (blue line) “C+ Betaine 0.6 M”, (orange line) “C+ Betaine 0.8 M”, (purple line) “C+ Betaine 1 M”, (green line) “C+ DMSO 5%”, (yellow line) “C+ DMSO 7.5%”; (**b**) Respective melt curves.

The temperature of the qLAMP assay was evaluated from 60 to 65 °C and the optimal amplification temperature resulted to be 64 °C as seen in figure S1 with a *T*_*t*_ of 15.9 ± 0.2 min for a sample of pure *D. polymorpha* DNA with a concentration of 567.3 ng µL-1. Regarding the addition of Betaine and DMSO, the best amplification results were obtained for the reactions without any supplements added, as described in Fig. 1a. The addition of DMSO promoted a significant increase in *T*_*t*_, which was concentration-dependent, being 27.9 ± 0.1 min for 5% DMSO and 42.3 ± 0.1 min for 7.5% DMSO. On the other hand, the supplementation with Betaine resulted in a *T*_*t*_ of approximately 20 min for all concentrations tested. From this experiment, it was also observed that supplementation with DMSO had an effect on the melting temperature, resulting in a shift of the melting temperature peak from 80.9 °C down to 76.6 °C depending on the concentration, as illustrated in Fig. 1b. The concentration of LB primer was also optimized, providing a faster qLAMP reaction with a *T*_*t*_ of 8.8 ± 0.2 min for the same sample of pure *D. polymorpha* DNA, without compromising the specificity of the reaction. The *T*_*t*_ values were obtained using the mathematical model previously described, being represented in Table S1and Table S2.

### Evaluation of primer specificity for qPCR and qLAMP

The specificity of the newly designed qPCR and qLAMP primers was firstly confirmed using BLAST and, later on, evaluated by testing a total of 63 environmental samples. The negative samples analyzed in this study included 17 well characterized species commonly found in the Guadalquivir River basin (12 different fish species, 1 crustacean, and 4 mollusks), up to four different individuals of each species, and five non-invaded water samples, making a total of 50 negative samples. For both qPCR and qLAMP methods, amplification was only observed for samples from *D. polymorpha* meat and for water samples contaminated with this species, proving the specificity of the designed primers. The positive samples analyzed included five samples from *D. polymorpha* meat and eight water samples from the Guadalquivir River. Two of these water samples corresponded to surface water from Los Bermejales reservoir, collected in December 2016, and from Iznájar reservoir, collected in May 2017. The other six water samples were river water in which the zebra mussels were transported, being tested as mixed water, after homogenizing the sample, and as settled water, after letting the particles settle down. Regarding the positive samples analyzed, the water sample from Los Bermejales reservoir was the only one in which no amplification neither by the qPCR approaches or the qLAMP method was observed. All the other positives samples showed amplification as expected, allowing the detection of *D. polymorpha*. A detailed list of the different species analyzed for specificity evaluation and the respective amplification results obtained for each method is described in Table 1.

**Table 1:** List of all samples analyzed for the specificity evaluation of both qPCR and qLAMP methods.

Sample	Standard international name	Nr. of samples	Sampling location	Sample provided by	qPCR		qLAMP
					Hydrolysis Probe	F3/B3	
**Negative samples**							
Fish							
* Luciobarbus sclateri*	Southern Iberian barbel	3	Guadalquivir river (Spain)	Guadalictio	–	–	–
* Pseudochondrostoma willkommii*	Southern straight-mouth nase	2			–	–	–
* Squalius alburnoides complex*	Calandino	3			–	–	–
* Squalius pyrenaicus*	Southern Iberian Chub	3			–	–	–
* Cobitis paludica*	Southern Iberian spined-loach	3			–	–	–
* Iberochondrostoma lemmingii*	Iberian arched-mouth nase	3			–	–	–
* Cyprinus carpio*	Carp	1			–	–	–
* Carassius gibelio*	Prussian carp	3			–	–	–
* Lepomis gibbosus*	Pumpkinseed	3			–	–	–
* Micropterus salmoides*	Largemouth Black-bass	3			–	–	–
* Gambusia holbrooki*	Eastern mosquitofish	3			–	–	–
* Alburnus alburnus*	Bleak	3			–	–	–
Mollusks							
* Corbicula fluminea*	Asian clam	1	Loire river (France)	USC	–	–	–
* Physa acuta*	Freshwater snail (general)	3	Guadalquivir river (Spain)	Guadalictio	–	–	–
* Ancylus fluviatilis*	Limpet (general)	3			–	–	–
fam. *Unionidae*	Freshwater mussel (general)	4			–	–	–
Crustacean							
* Procambarus clarkii*	red swamp crayfish	1	Guadalquivir river (Spain)	Guadalictio	–	–	–
Negative water samples							
Lima river	*	1	Lima River (Portugal)	INL	–	–	–
Ave river		1	Ave River (Portugal)		–	–	–
Estuary of lima river		1	Estuary of Lima River (Portugal)		–	–	–
Fountain water		1	Fountain water (Portugal)		–	–	–
Lake water		1	Lake water (Portugal)		–	–	–
**Positive samples**							
Meat samples							
* D. polymorpha* 1	Zebra mussel	1	Guadalquivir river (Spain)	Confederación Hidrográfica del Guadalquivir	+	+	+
* D. polymorpha* 2		1			+	+	+
* D. polymorpha* 3		1			+	+	+
* D. polymorpha* 4		1			+	+	+
* D. polymorpha* 5		1			+	+	+
Positive water samples							
* D.p.* transport water 1	*	1	Guadalquivir river (Spain)	Confederación Hidrográfica del Guadalquivir	+	+	+
* D.p.* transport water 2		1			+	+	+
* D.p.* transport water 3		1			+	+	+
* D.p.* transport water 4		1			+	+	+
* D.p.* transport water 5		1			+	+	+
* D.p.* transport water 6		1			+	+	+
Surface river water 1 (Los Bermejales, Dec 2016)		1			–	–	–
Surface river water 2 (Iznájar, May 2017)		1			+	+	+

*Water samples with natural eDNA.

### Evaluation of sensitivity for qPCR and qLAMP

**Figure 2:**
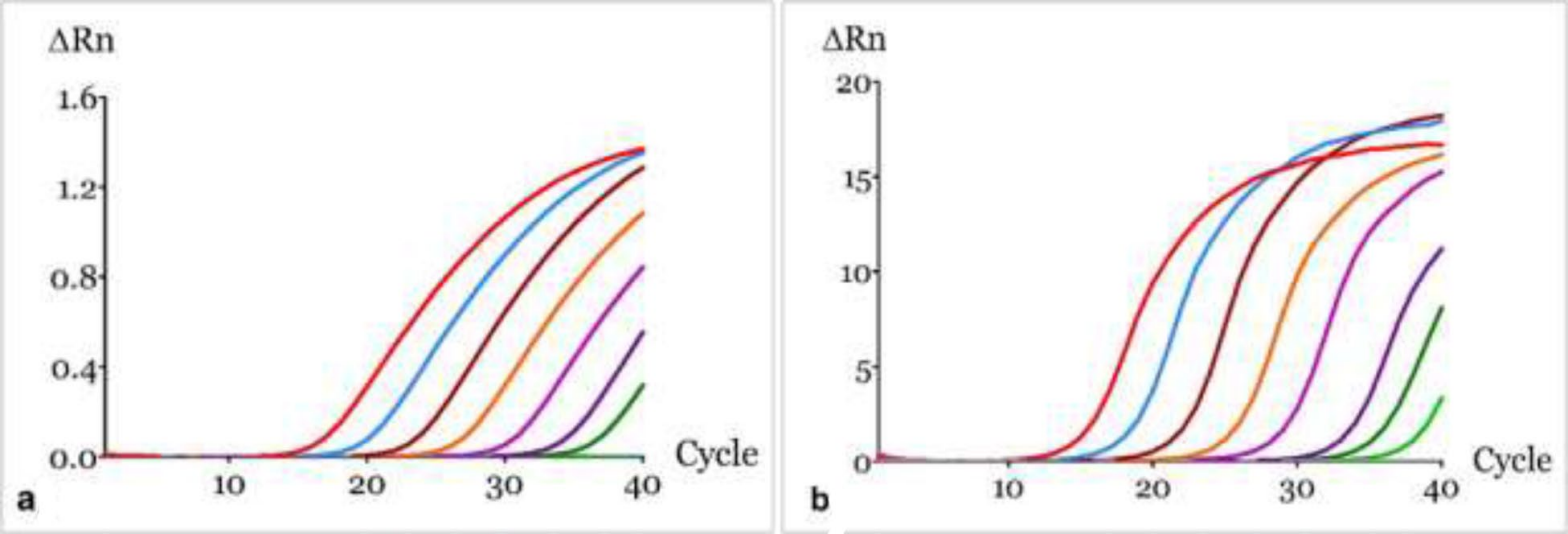
Sensitivity evaluation through the analysis of ten-fold serial dilutions (Dil.1 – Dil.10) of pure *D. polymorpha* DNA with initial concentration of 567.30 ng/µL performed using GraphPad Prism version 8.0.0 for Windows, GraphPad Software, San Diego, California USA, www.graphpad.com: (red line) Dil.1, (light blue line) Dil.2, (deep red) Dil.3, (orange) Dil.4, (fucsia line) Dil.5, (purple line) Dil.6, (dark blue line) Dil.7, (dark green line) Dil.8, (green line) Dil.9, (yellow line) Dil.10. (**a**) qPCR method with hydrolysis probe; (**b**) qPCR method with F3/B3 primers. ΔRn is the magnitude of the signal generated, being given by the normalized reporter (Rn) subtracted by the baseline.

**Figure 3:**
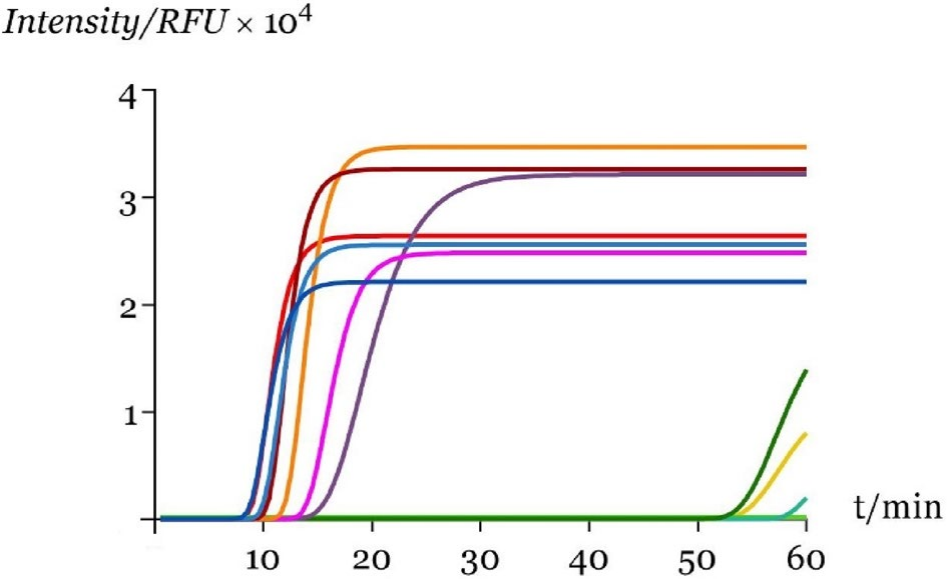
Sensitivity evaluation of the qLAMP method through the analysis of ten-fold serial dilutions (Dil.1 – Dil.10) of pure *D. polymorpha* DNA with initial concentration of 567.30 ng/µL according to the fittings from Eq. (1) performed using GraphPad Prism version 8.0.0 for Windows, GraphPad Software, San Diego, California USA, www.graphpad.com: (dark blue line) Dil.0, (red line) Dil.1, (light blue line) Dil.2, (deep red line) Dil.3, (orange line) Dil.4, (fucsia line) Dil.5, (purple line) Dil.6, (blue line) Dil.7, (dark green line) Dil.8, (green line) Dil.9, (yellow line) Dil.10.

The sensitivity of the amplification methods was evaluated through the analysis of ten-fold serial dilutions of pure *D. polymorpha* DNA. For both qPCR approaches, positive detection was obtained from 567.3 ng µL^−1^ (Dil.0) down to 0.056 pg µL^−1^ (Dil.7), covering a dynamic range of seven orders of magnitude. Regarding the qLAMP method, positive detection was obtained from 567.3 ng µL^−1^ (Dil.0) down to 0.56 pg µL^−1^ (Dil.6), covering a dynamic range of six orders of magnitude. This corresponds to 3.992 × 10^5^ copies µL^−1^ for both qPCR methods and 2.010 × 10^6^ copies µL^−1^ for the qLAMP method^39^. All samples were tested in duplicate and the average amplification results obtained for the sensitivity evaluation of these methods are illustrated in Figs. 2 and 3, respectively.

### Mathematical modeling

**Figure 4:**
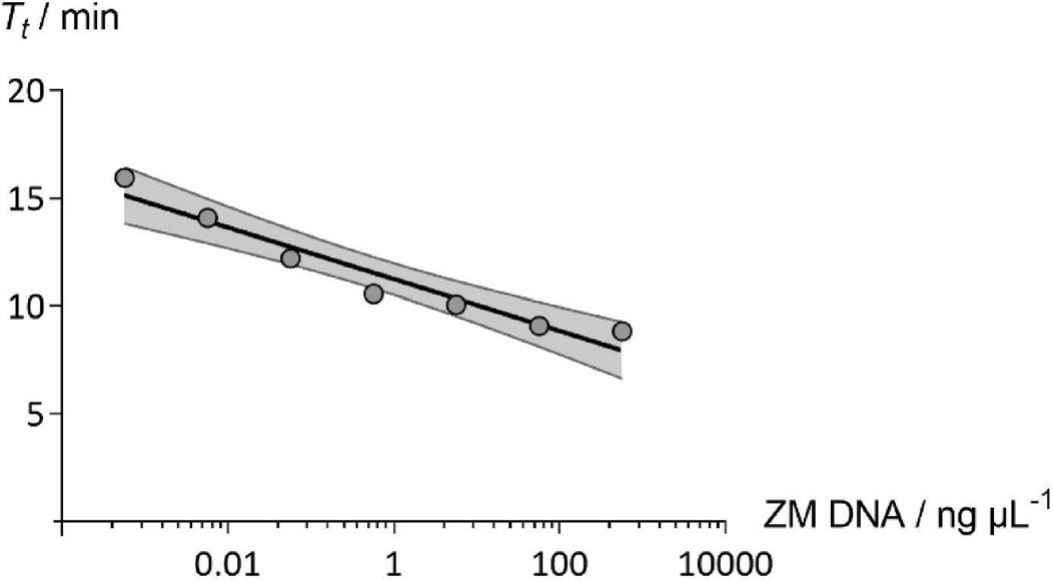
LOD for qLAMP in pure *D. polymorpha* DNA samples. Gray lines represent the 95% confidence bands for the linear estimation performed using GraphPad Prism version 8.0.0 for Windows, GraphPad Software, San Diego, California USA, www.graphpad.com.

Statistical analysis of the fittings indicated the suitability of the proposed equation (Eq. 1) for modeling qLAMP kinetics (Table S1 and Table S2). In all samples, the fitted models were statistically significant, with p-values from the Fisher’s F-test lower than 0.001 and showed an excellent correlation with the experimental data (Figure S1 and Figure S2), having extremely high adjusted coefficients of determination ($$r_{adj}^{2} > 0.999$$). The estimated parameters $$ RF_{{s_{max} }}$$, $$ \mu_{max}$$ and *T*_*t*_ were also statistically significant (Student t-test; α = 0.05) in all amplified samples (whenever one of the replicates failed to amplify, the sample was considered negative). *T*_*t*_ was the parameter that exhibited more sensitivity to changes both in the type and concentration of supplements in the master mix, and to template DNA concentration. The linear range between pure *D. polymorpha* DNA (567.3 ng µL^−1^) and dilution six (0.56 pg µL^−1^), with *T*_*t*_ values ranging from 8.8 ± 0.2 min to 16.0 ± 0.2 min, is described in Fig. 4.

## Discussion

Due to the fast spreading of *D. polymorpha*, an early detection of this highly invasive species is a critical point for a more efficient management and control, making the development of methods to detect this species at low densities a high priority. The analysis of eDNA has proven to be a more effective detection method for low population densities, especially in the case of aquatic invasive species^21,22,40^. DNA amplification using PCR is the most widely adopted method for DNA analysis due to its high sensitivity and highly efficient exponential amplification. Despite of its advantages, this technique also has limitations, which promoted the development of alternative isothermal amplification methods such as LAMP. When compared to PCR, LAMP-based methods have higher tolerance to the presence of inhibitors, are performed at constant temperature, not requiring expensive equipment, and allow naked-eye detection, thus simplifying its implementation in point-of-need detection devices^41^. Several PCR methods have been developed to detect *D. polymorpha* through the analysis of eDNA^26,42,43^. However, to our knowledge, only one method has been published regarding the detection of this species using LAMP^44^, more studies being required in order to better evaluate the performance of this technique for the detection of invasive species in environmental samples.

In this study, a novel qLAMP method was developed and evaluated for the detection of *D. polymorpha* in the Guadalquivir River basin, and compared with a qPCR method, also developed in this work, using two different detection chemistries. The qLAMP method was optimized regarding primers concentration, amplification temperature and supplement addition. The optimal temperature obtained was 64 °C and the effect of supplement addition was evaluated by testing Betaine and DMSO. Betaine equalizes the contribution of G-C and A-T base pairing while DMSO disrupts base pairing, facilitating strand separation by interfering with hydrogen bonding^45^. Both agents are commonly used to improve LAMP sensitivity and specificity, reducing false positive results^46^. The reaction was supplemented with these agents in different concentrations in order to evaluate their effect. As described in Fig. 1a), the supplement addition resulted in lower LAMP efficiency, especially regarding the supplementation with DMSO, which increased the threshold time from 16 min up to 42 min. Since the addition of these agents did not improve efficiency and considering that no false positive results were obtained for any of the samples analyzed, the qLAMP method developed was performed without supplements. In addition, by optimizing the concentration of LB primer, the threshold time was reduced from 16 to 9 min, allowing a faster amplification without negatively affecting the specificity of the reaction. For the qPCR method using the hydrolysis probe for detection, the optimization was focused on primers and probe concentration, and annealing temperature. For this approach the optimal temperature resulted to be 62 °C as seen in table S3. For a better comparison between the qLAMP and qPCR methods, a qPCR using F3/ B3 primers and an intercalating dye was also evaluated, being performed with an annealing temperature of 60 °C as indicated in the manufacturer’s protocol.

The qLAMP method and both qPCR approaches were evaluated regarding primers and probe specificity. From the 63 environmental samples analyzed, amplification was only observed for positive samples, *D. polymorpha* meat and contaminated water samples. Therefore no false positives were obtained, proving the specificity of primers and probe, as described in Table 1. However, there was one positive water sample in which this species could not be detected neither by the qLAMP method nor the qPCR approaches. This sample corresponded to surface water collected from Los Bermejales reservoir (Guadalquivir River basin, Spain), which is known to be invaded by zebra mussels. There is a relation between the amount of eDNA and the species density, however the sampling strategy strongly influences the amount of eDNA in the samples because sampling can be done close to the species or far away from it. In addition, the degradation and dilution of eDNA is influenced by several factors, especially under natural conditions^18^. Another surface water sample was collected from Iznájar reservoir (Guadalquivir River basin, Spain) but, in this case, the presence of *D. polymorpha* was detected by all the methods tested. However, it is also important to consider that the surface water sample from Los Bermejales reservoir was collected in December, while the one from Iznájar reservoir was collected in May, which corresponds to a more active season for this species due to reproduction, thus facilitating its detection^16^. Zebra mussels generally reproduce when the water temperature is above 12 °C, usually during spring or summer^3,16,47^.

Regarding sensitivity, both detection chemistries used for qPCR were more sensitive than the qLAMP approach by one order of magnitude. In terms of speed, detection was achieved in about 9 min with qLAMP, while with the qPCR methods 23 min were needed using detection with hydrolysis probe and 29 min were needed using primers F3/ B3 and the intercalating dye. Therefore, the qLAMP method proved to be faster than both qPCR approaches, being one of the advantages of this isothermal amplification method. These DNA-based methods are both promising alternatives to the traditional microscopic analysis of the water, which is very labor intensive and time consuming.

In conclusion, a qLAMP method for the detection of *D. polymorpha* in environmental water samples was developed and compared with two qPCR methods using different detection chemistries. The three methods evaluated exhibited similar results in terms of specificity, allowing the detection of this species with no false positives. Regarding sensitivity, the qPCR approaches achieved better results by one order of magnitude compared to qLAMP. On the other hand, the qLAMP method proved to be much faster than both qPCR approaches, which together with its simplified thermal control, and the possibility of implementing naked-eye detection, makes this a very promising method for decentralized field analysis.

In order to improve the detection of *D. polymorpha* and other invasive species, further work should be done to better understand the effect of environmental and sampling conditions (e.g. season, sampling depth, water temperature) on the amount of target eDNA present in the water samples, and on the integration of detection methods with a pre-concentration step in order to move towards *on-site* early detection of invasive species that will surely contribute to the development of early warning systems for the presence of IAS.

## Methods

### Samples

Water samples from different locations including negative samples from Lima and Ave rivers as well as a fountain and a lake water sample all from Portugal, and positive samples such as transport water from *D. polymorpha* specimen and water from Los Bermejales and Iznajar reservoirs as well as samples from different species particularly from twelve fish species, as well as three mollusks and one crustacean species all from the Guadalquivir river provided by the company Guadalictio, except from one of the mollusks species coming from the Loire river and provided by the University of Santiago de Compostela. Additionally, five well identified *D. polymorpha* samples were kindly provided by Confederación Hidrogáfica del Guadalquivir.All referred samples were analyzed for the specificity evaluation of both qPCR and qLAMP approaches developed in this study. Consequently, a total of 63 samples were tested. The main goal of this evaluation was to ensure no false positive signal from other organisms, especially from those that could potentially be present in the Guadalquivir River basin. A detailed list of all the samples analyzed in this study is provided in Table 1.

### DNA extraction

DNA extraction and purification from the previously mentioned samples was performed with NucleoSpin Food Kit (Macherey–Nagel, Düren, Germany) with two modifications to the protocol. These modifications were: first, the addition of 10 µL of RNase A (10 mg mL^−1^) in the lysis step, which was performed at 65 °C for 1 h with continuous agitation (1200 rpm), and, second, the elution was performed in two steps, adding 50 µL of Buffer CE (preheated at 70 °C) at each time. After extraction and purification, total DNA quantification was performed for each sample using the spectrophotometer NanoDrop 2000 (Thermo Scientific , Waltham, MA, United States).

### Gene selection and primers design for qPCR and qLAMP

**Figure 5:**
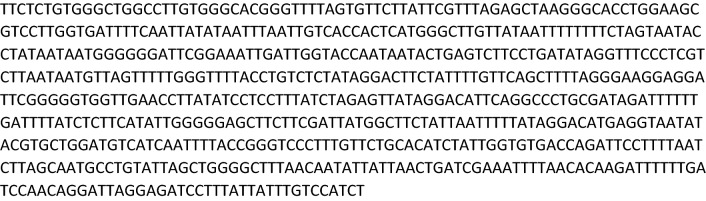
Consensus sequence obtained from the alignment of 40 COI gene sequences from *D. polymorpha* retrieved from GenBank, and aligned with the CLC Sequence Viewer 7.7 software (CLC Bio-QIAGEN, Aarhus, Denmark).

The detection of *D. polymorpha* was accomplished by targeting a specific region of the mitochondrial gene cytochrome C oxidase subunit I (COI). This gene has been previously used to identify *D. polymorpha* as well as other species, such as *Dreissena bugensis*, *Limnoperna fortunei* and *Eriocheir sinensis*^42,48,49^. A total of 40 genetic sequences for *D. polymorpha* COI gene were obtained from the NCBI GenBank and aligned with the CLC Sequence Viewer 7.7 software (CLC Bio-QIAGEN, Aarhus, Denmark) in order to determine a consensus sequence displayed in Fig. 5, for primer design. Primers design for the qLAMP method was performed by uploading the consensus sequence to the free software Primer Explorer V4^50^. For the qPCR method, primers were designed with the free online software Primer3^51^ and a hydrolysis probe was also designed for this approach. The specificity of the newly designed primers and probe was verified in silico using the Basic Local Alignment Search Tool (BLAST)^52^ BLAST reference number (RID) is indicated for each case in Table 2, and, later on, in vitro by analyzing the environmental samples previously described. All primers and probe were purchased from Integrated DNA Technologies Inc. (IDT, Leuven, Belgium). Detailed information regarding these oligonucleotides is described in Table 2.

**Table 2:** Primers and probe sequences for qPCR and qLAMP methods.

Detection method	Primer description		Sequence (5′ → 3′)	BLAST reference number (RID)
qPCR	Forward Primer	Dp-F	TAACAAGCCCATGAGTGGTGAC	71137B8A016
	Reverse Primer	Dp-R	TCTCTGTGGGCTGGCCTTGT	7116FN1E016
	Hydrolysis Probe	Dp-P	FAM/ACCAAGGAC/ZEN/GCTTCCAGGTGCC/IABkFQ	711P29A2013
qLAMP	Forward Inner Primer	Dp-FIP	ACAAGCCCATGAGTGGTGACAAT-ACCTGGAAGCGTCCTTG	711EB351016&&&711GP1Z1013
	Backward Inner Primer	Dp-BIP	AATGGGGGGATTCGGAAATTGATTGGTA-AACTAACATTATTAAGACGAGGGAAAC	711J8VHK016&&&711M58MU013
	Forward outer primer	Dp-F3*	GGCACGGGTTTTAGTGTTCT	7118TZCA016
	Backward outer primer	Dp-B3*	ACAAAATAGAAGTCCTATAGAGACAG	711AACAG013
	Backward Loop primer	Dp-LB	CCAATAATACTGAGTCTTCCTGA	711BJ6D1016

*F3/B3—outer primers, also used for qPCR with Sybr Green I; FIP/BIP—inner primers.

### qPCR

Two different qPCR approaches were tested in order to have a better comparison with the qLAMP method developed in this study. The first approach was performed using newly designed primers for qPCR and a hydrolysis probe, while the second one was performed using the forward and backward outer primers (F3 and B3) designed for the qLAMP method and an intercalating dye.

The primers designed for the qPCR method including a hydrolysis probe amplified a 128 bp DNA fragment with of the mitochondrial COI gene. The final reaction volume was 20 µL, with 2 µL of template DNA. In this approach, 10 µL of Maxima Probe/ROX qPCR Master Mix (Thermo Fisher Scientific Inc., Waltham, MA, USA) were used for each reaction. The reaction was optimized regarding primers and probe concentration, and amplification temperature, which was evaluated from 60 to 65 °C. The concentrations of primers and probe used were 100 nM and 150 nM, respectively. The thermal profile included an initial Uracil-DNA Glycosylase (UDG) treatment at 50 °C for 2 min and a hot start polymerase activation at 95 °C for 10 min, followed by 40 cycles of dissociation at 95 °C for 15 s and annealing-extension at 62 °C for 30 s. All experiments were carried out in a StepOne Plus Real-Time PCR system (Applied Biosystems, Foster City, CA, USA) with StepOne Software v2.1.

The second approach involved the use on an intercalating dye, in this case the F3/ B3 primers designed for qLAMP were used as primers for the qPCR, therefore targeting the same DNA fragment as the LAMP method (254 bp). The reactions were performed as detailed above but the Master Mix was replaced by Maxima SYBR Green/ ROX qPCR (Thermo Fisher Scientific Inc., Waltham, MA, USA), the primer concentration was 200 nM and the thermal profile included the UDG treatment at 50 °C for 2 min and a hot start at 95 °C for 10 min, followed by 40 cycles of dissociation at 95 °C for 15 s and annealing-extension at 60 °C for 1 min two-step cycling protocol as detailed in manufacturer’s product information. A melt curve step was included after the qPCR amplification, consisting on heating up to 95 °C for 15 s, cooling down to 60 °C for 1 min and heating up to 95 °C for 15 s, acquiring fluorescence every 0.3 °C.

### Evaluation of qPCR methods

Specificity and sensitivity of both qPCR approaches were evaluated. The specificity evaluation was performed by testing the 63 environmental samples described in Table 1. The sensitivity of the methods was evaluated using ten-fold dilutions, starting from a solution of pure *D. polymorpha* DNA exhibiting a concentration of 567.3 ng µL^−1^ which corresponds to approximately 3.992 × 10^5^ copies µL^−1^. All samples were tested in duplicate.

### qLAMP

The newly designed LAMP primers amplified a 254 bp DNA fragment of the COI gene. All qLAMP reactions were performed with a sample volume of 2 µL to achieve a final volume of 20 µL. For each reaction, 10 µL of GspSSD Isothermal Master Mix ISO-001 (OptiGene Ltd., Horsham, UK) were used. The reaction was optimized attending to primers concentration, amplification temperature and reaction supplementation with Betaine and dimethyl sulfoxide (DMSO) (Sigma-Aldrich, Darmstadt, Germany). The amplification temperature was evaluated in the 60 °C to 65 °C range for optimization. Regarding reaction supplements, Betaine was tested at 0.6 M, 0.8 M and 1 M, while DMSO was tested at 5% and 7.5%.

The concentration of primers used was 1600 nM for FIP/BIP, 200 nM for F3/B3 and 800 nM for LB, being DNA amplification of the environmental samples performed at 64 °C for 1 h without supplements. In addition, a melt curve step was included in all qLAMP experiments, consisting of heating up to 98 °C for 15 s, cooling down to 75 °C for 1 min and heating up to 95 °C for 15 s, acquiring fluorescence every 0.3 °C.

### Evaluation of qLAMP method

Sensitivity and specificity evaluation was also performed for this method in order to compare with the qPCR approaches. All samples were tested in duplicate and a sample was only considered as positive when both replicates were amplified with a threshold time (*T*_*t*_) lower than 30 min.

### Mathematical modeling

The qLAMP reaction was performed in a regular qPCR thermocycler (StepOne Plus Real-Time PCR). However, the equipment’s software, which is not specific for this technique, led to infra- or overestimation errors in the determination of *T*_*t*_ values. For this reason, the *T*_*t*_ values were calculated from the raw data by mathematical modeling the qLAMP kinetics, following an approach similar to that described by Garrido-Maestu et al*.*^35^. In this case, the increase of fluorescence signal (*RF*) as a function of time (*t*) was modeled as the sum of two equations:

1 $$ RF\left( t \right) = { }RF_{ns} \left( t \right) + { }RF_{s} \left( t \right) $$

being $RF_{ns} \left( t \right)$ a pseudo-first order equation that accounted for the non-specific fluorescence signal (e.g. primer binding to non-target DNA):

2 $$ RF_{ns} \left( t \right) = RF_{bg} + { }RF_{{ns_{max} }} \left( {1 - e^{{ - k_{ns} { }t}} } \right) $$

where $RF_{bg}$ represented the relative fluorescence background, $RF_{{ns_{max} }}$ was the maximum non-specific fluorescence signal, and $k_{ns}$ was the non-specific fluorescence increase rate (min^−1^), and being $RF_{s} \left( t \right)$ a sigmoid equation (reparametrized Gompertz), which accounted for the target DNA amplification:

3 $$ RF_{s} \left( t \right) = RF_{{s_{max} }} e^{{ - e^{{1 + \frac{{e{ }\mu_{max} { }\left( {T_{t} - t} \right)}}{{RF_{{s_{max} }} }}}} }} $$

where $RF_{{s_{max} }}$ represented the maximum specific fluorescence signal, $\mu_{max}$ was the maximum specific amplification rate (min^−1^) and *T*_*t*_ (min) represented the threshold time at which the fluorescence intensity of the amplified DNA becomes statistically significantly higher than the background signal.

Data fitting, assessment of the model parameters significance (Student t-test; α = 0.05), and consistency of the mathematical model (Fisher’s F test; *p* < 0.05) were performed with *Mathematica 9* (Wolfram Research, Inc.).

## Supplementary Information

Supplementary Information.
