# Supplementary material for: Faster monitoring of the invasive alien species (IAS) Dreissena polymorpha in river basins through isothermal amplification
Source: Sci Rep. 2021 May 13;11:10175. doi: 10.1038/s41598-021-89574-w (PMC8119715; doi:10.1038/s41598-021-89574-w)
Supplement: Supplementary file 1 — Supplementary Information. [file 41598_2021_89574_MOESM1_ESM.pdf]

Supplementary Information:

Title: Faster monitoring of the Invasive Alien Species (IAS) *Dreissena polymorpha* in river basins through isothermal amplification

Authors and Affiliations

Joana Carvalho[1,2], Alejandro Garrido-Maestu[1], Sarah Azinheiro[1,2], Pablo Fuciños[1], Jorge Barros-Velázquez[2], Ramón J. De Miguel[3,4], Verónica Gros[5], Marta Prado[1*]

[1] International Iberian Nanotechnology Laboratory (INL), Portugal

[2] Department of Analytical Chemistry, Nutrition and Food Science, School of Veterinary Sciences, University of Santiago de Compostela, Spain

[3]Department of Zoology, University of Cordoba, Spain

[4]Guadalictio S.L., Spain

[5]Confederación Hidrográfica del Guadalquivir. Ministerio para la Transición Ecológica y el Reto Demográfico, Spain

Corresponding author

E-mail: marta.prado@inl.int

Postal Address: Food Quality and Safety Research Group. International Iberian Nanotechnology Laboratory (INL) Av. Mestre José Veiga s/n 4715-330 Braga – Portugal

Phone Number: + 351 253 140 112

Figure S1: Result of optimal amplification temperature for qLAMP method for *D. polymorpha*

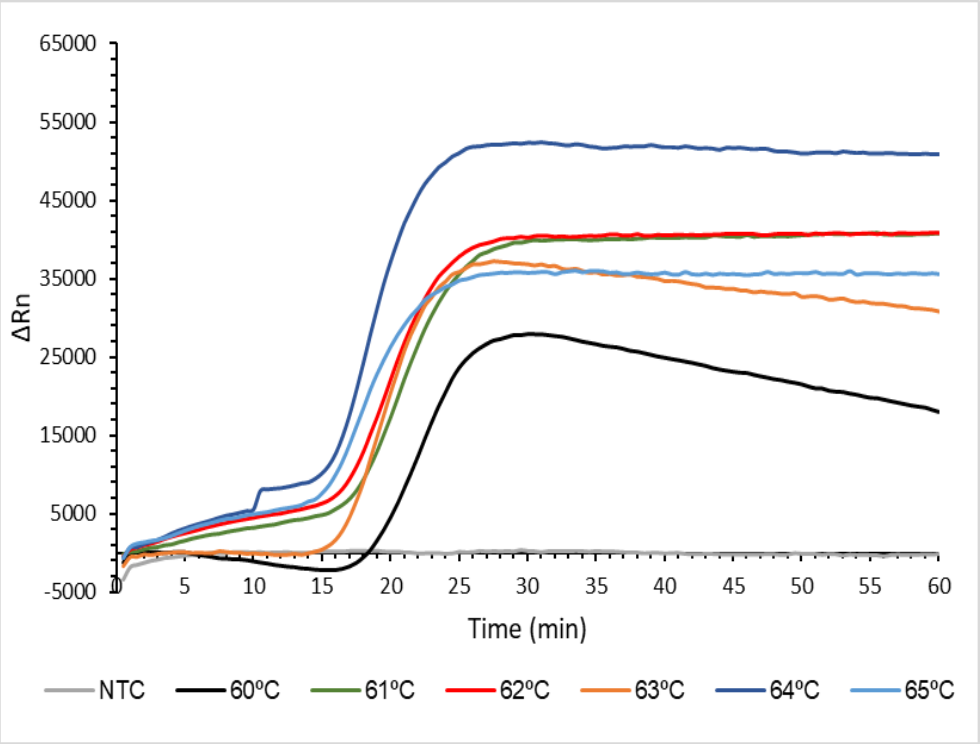

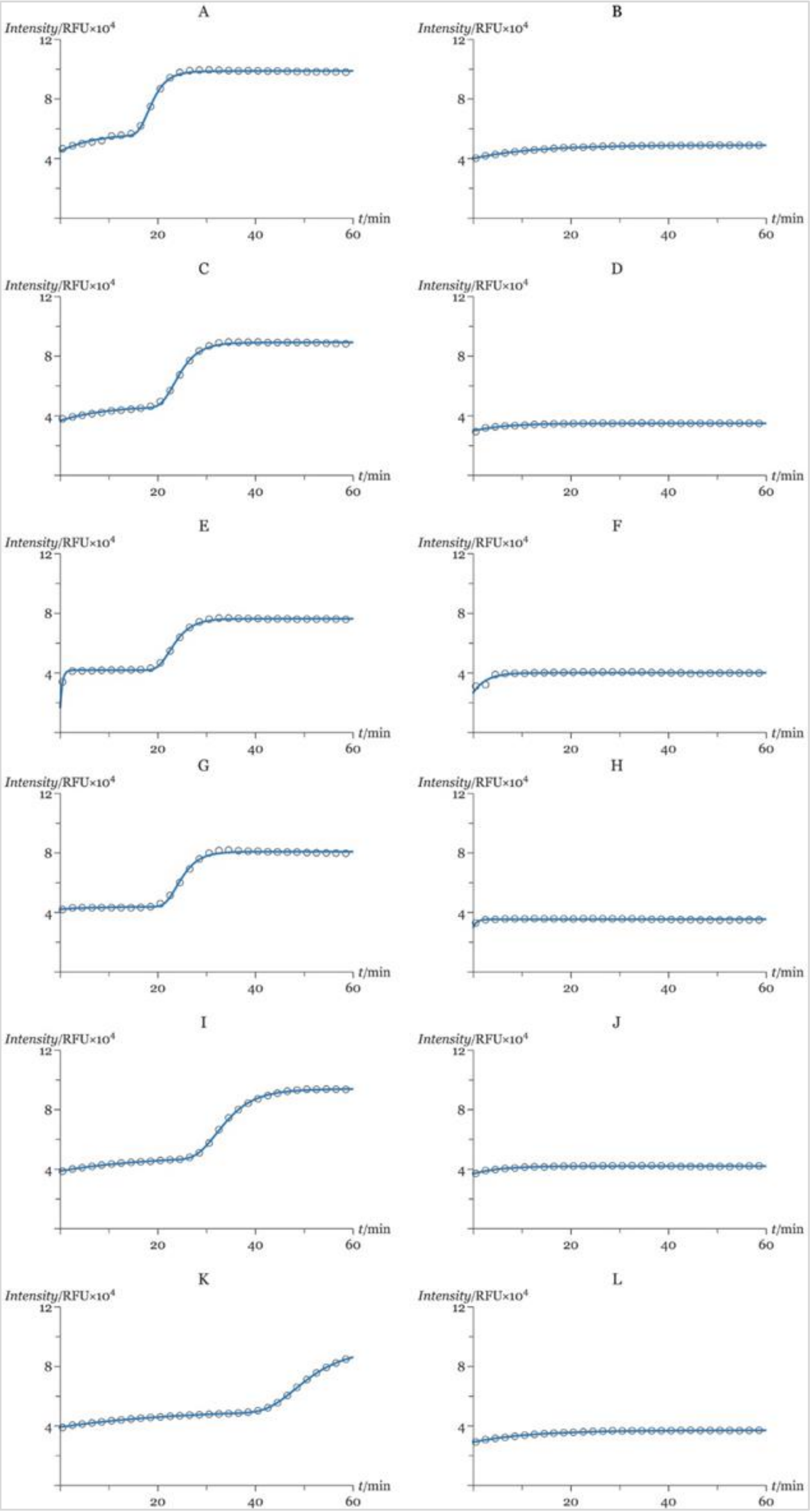

Figure S2. qLAMP amplification kinetics in pure *D. polymorpha* DNA (C+) supplemented with different additives. **A:** "C+ No Supplement", **B:** "NTC No Supplement", **C:** "C+ 0.6 M betaine", **D:** "NTC 0.6 M betaine", **E:** "C+ 0.8 M betaine", **F:** "NTC 0.8 M betaine", **G:** "C+ 1 M betaine", **H:** "NTC 1 M betaine", **I:** "C+ 5 % DMSO", **J:** "NTC 5 % DMSO", **K:** "C+ 7.5 % DMSO", **L:** "NTC 7.5 % DMSO". Symbols: experimental data. Lines: model fittings according to Eq. (1).

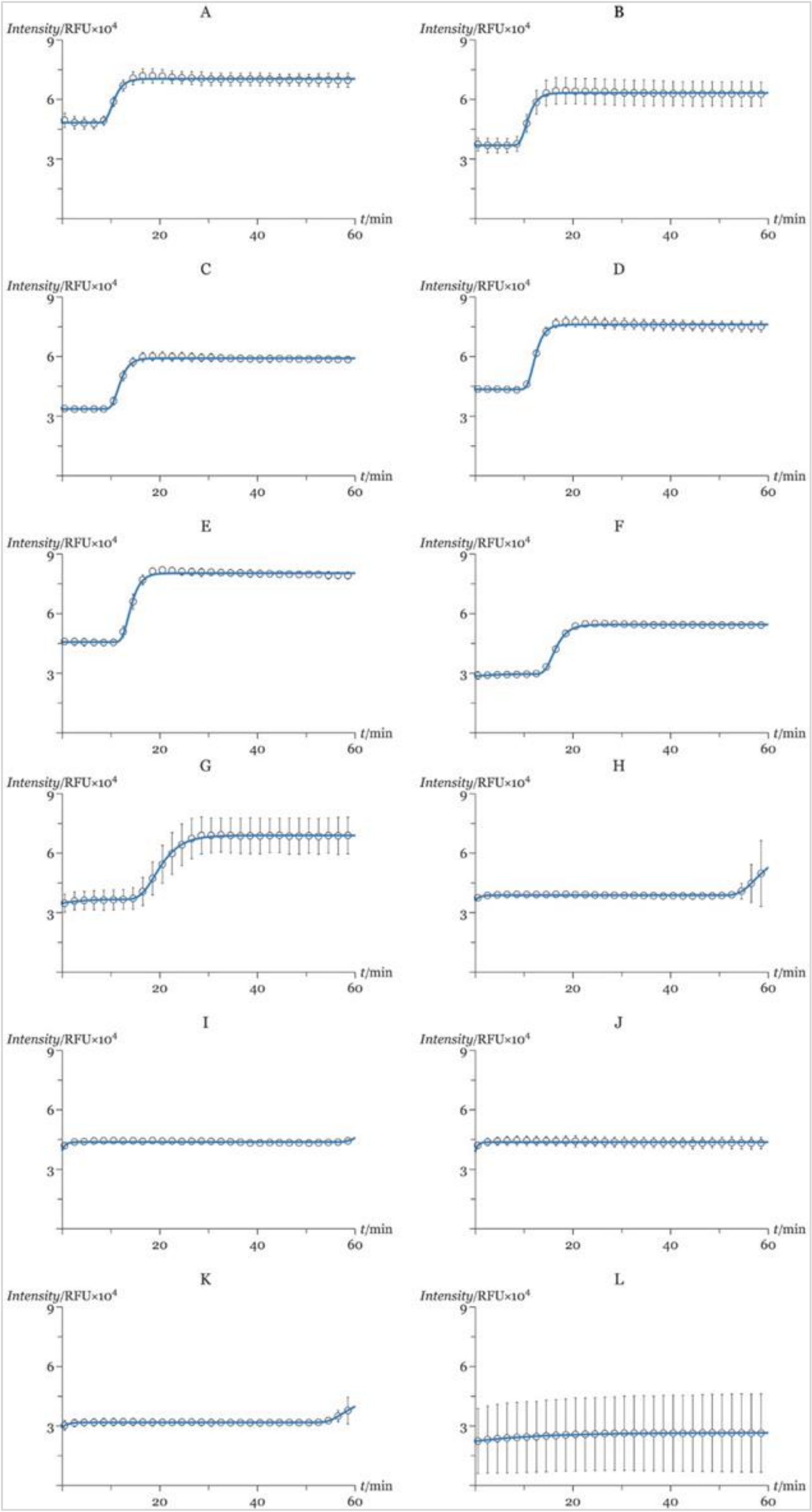

Figure S3. qLAMP amplification kinetics in ten-fold serial dilutions (Dil.1 – Dil.10) of pure *D. polymorpha* DNA (567.3 ng/µL). **A:** pure *D. polymorpha* DNA (567.3 ng/µL), **B:** Dil.1, **C:** Dil.2, **D:** Dil.3, **E**: Dil.4, **F:** Dil.5, **G:** Dil.6, **H:** Dil.7, **I:** Dil.8, **J:** Dil.9, **K:** Dil.10, **L:** NTC. Symbols: experimental data. Lines: model fittings according to Eq. (1).

Table S1. Parametric estimations and Goodness of Fit analysis of the qLAMP amplification model (Eq. 1) applied to DNA samples from zebra mussel meat supplemented with different additives.

Sample & Supplement	Best-Fit parameters						Goodness of Fit		
	BG	***NTRF$_{max}$***	***kNT* (min^{-1})**	***RF$_{max}$***	**μ_{max} (min^{-1})**	**T_t (min)**	r^2_{adj}	***F*-ratio**	***p*-value**
C+ No Supplement	44966.6±1003.2	12248.0±1441.6	0.137±0.045	41756.4±1587.1	7352.36±321.37	15.88±0.19	0.9999	281761.0	< 0.001
NTC No Supplement	40082.9±108.5	8955.8±100.9	0.084±0.002	605.2±0.0	18.25±0.00	158.43±0.00	1.0000	3172163.3	< 0.001
C+ 0.6 M betaine	36767.9±766.0	10041.6±1104.3	0.106±0.034	42572.4±1225.5	5760.54±185.19	20.60±0.18	0.9999	279807.7	< 0.001
NTC 0.6 M betaine	29979.2±211.5	5007.6±205.6	0.147±0.010	1080.6±0.0	14.90±0.00	397.05±0.00	1.0000	750986.7	< 0.001
C+ 0.8 M betaine	17096.6±8948.2	24799.4±8923.3	2.299±0.627	34559.1±196.7	5077.45±149.95	19.88±0.12	1.0000	416831.8	< 0.001
NTC 0.8 M betaine	26983.5±1342.4	13215.6±1330.6	0.365±0.050	1795.9±0.0	14.36±0.00	380.94±0.00	0.9997	75348.3	< 0.001
C+ 1 M betaine	42202.7±1130.1	1722.6±1010.2	*0.150±0.275	36967.5±940.5	5527.80±275.31	21.37±0.23	0.9999	142144.2	< 0.001
NTC 1 M betaine	*29900.1±4.27·10^6	5000.1±2935.2	1.435±0.947	*30000.0±3.12·10^9	*7.56±5.45·10^5	*602.26±5.30·10^7	0.9999	203061.5	< 0.001
C+ 5% DMSO	38810.7±267.6	10378.1±857.6	0.056±0.010	45207.8±700.5	4019.21±46.03	27.85±0.11	1.0000	1296396.6	< 0.001
NTC 5% DMSO	36249.5±890.6	5111.7±341.8	0.184±0.026	10900.2±58.8	*0.00±10.28	188.70±0.00	1.0000	557417.7	< 0.001
C+ 7.5% DMSO	39237.7±97.4	12300.5±262.5	0.040±0.002	40566.2±356.0	2600.37±17.37	42.31±0.06	1.0000	4338509.8	< 0.001
NTC 7.5% DMSO	28756.8±3864.4	7982.7±384.2	0.084±0.007	6518.9±255.0	*0.00±16.64	488.10±0.00	1.0000	942678.5	< 0.001

*Not significant parameter (Student *t*-test; α = 0.05).
Best-Fit parameters are expressed as Estimate ± Confidence Interval (α = 0.05).

Table S2. Parametric estimations and Goodness of Fit analysis of the qLAMP amplification model (Eq. 1) applied to DNA samples from zebra mussel (ZM) meat at different concentrations.

Sample & Supplement	Best-Fit parameters						Goodness of Fit		
	BG	***NTRF$_{max}$***	***kNT* (min^{-1})**	***RF$_{max}$***	**μ_{max} (min^{-1})**	**T_t (min)**	r^2_{adj}	***F*-ratio**	***p*-value**
ZM meat 1	48332.9±1100.0	*0.0±1448.4	0.385±0.000	22094.0±546.1	6381.5±617.9	8.83±0.21	0.9999	191494.7	< 0.001
ZM meat 1 - D1	36972.5±827.1	*0.0±1141.1	0.328±0.000	26383.0±464.1	7685.2±504.0	9.07±0.14	0.9999	231866.6	< 0.001
ZM meat 1 - D2	33565.8±1134.1	*78.6±943.5	*0.330±12.138	25568.1±807.9	7340.7±427.3	10.05±0.14	0.9999	265583	< 0.001
ZM meat 1 - D3	43523.8±357.2	60.3±0	*0.000±0.220	32616.0±598.4	9717.0±766.9	10.56±0.15	0.9999	159383.1	< 0.001
ZM meat 1 - D4	45716.7±1703.4	*0.0±1465.5	*0.326±4·10^{13}	34707.6±892.8	9446.3±632.8	12.19±0.16	0.9999	185034.1	< 0.001
ZM meat 1 - D5	28569.4±552.0	1125.0±500.0	*0.209±0.305	24816.9±519.1	5392.9±184.5	14.07±0.11	1.0000	490970.3	< 0.001
ZM meat 1 - D6	34770.9±718.1	2096.4±619.2	0.226±0.193	32125.1±499.9	3990.1±105.6	15.95±0.16	1.0000	439283.5	< 0.001
ZM meat 1 - D7	36368.8±1519.6	2487.7±1514.6	1.065±0.700	20809.9±2997.2	2477.3±110.4	#54.08±0.16	1.0000	402766.1	< 0.001
ZM meat 1 - D8	39917.0±2880.9	3932.8±2874.7	1.392±0.994	*5324.8±27016.3	*1081.0±2116.5	#58.18±3.14	0.9999	279048.8	< 0.001
ZM meat 1 - D9	39097.7±5115.0	*4515.1±5175.4	1.843±1.843	2694.4±337.5	*0.0±9.4	#148.73±0	0.9999	223914.4	< 0.001
ZM meat 1 - D10	29374.6±611.6	2412.7±609.3	0.952±0.269	12009.6±1419.6	1519.5±55.5	#54.45±0.13	1.0000	1260656	< 0.001
NTC	22298.0±101.4	4343.3±92.1	0.071±0.004	*723.7±9.1·10^{6}	*18.3±4.7·10^{8}	*87.23±7.15·10^{8}	1.0000	955882.3	< 0.001

*Not significant parameter (Student *t*-test; α = 0.05).
#At least one replicate with no amplification
Best-Fit parameters are expressed as Estimate ± Confidence Interval (α = 0.05).

Table S3. Ct values resulting from the optimization of the qPCR method using the hydrolysis probe

Sample	Ct
60C	13.6
61C	13.1
62C	10.3
63C	11.8
64C	11.7
65C	11.7
